# Interactions between Metabolic Syndrome, MASLD, and Arterial Stiffening: A Single-Center Cross-Sectional Study

**DOI:** 10.3390/healthcare11192696

**Published:** 2023-10-09

**Authors:** Adelaida Solomon, Mihai Octavian Negrea, Călin Remus Cipăian, Adrian Boicean, Romeo Mihaila, Cristina Rezi, Bianca Andreea Cristinescu, Cristian Stefan Berghea-Neamtu, Mirela Livia Popa, Minodora Teodoru, Oana Stoia, Bogdan Neamtu

**Affiliations:** 1 Faculty of Medicine, “Lucian Blaga” University, 550024 Sibiu, Romania; solomonadelaida@gmail.com (A.S.); calin.cipaian@ulbsibiu.ro (C.R.C.); adrian.boicean@ulbsibiu.ro (A.B.); romeo.mihaila@ulbsibiu.ro (R.M.); cristina.rezi@ulbsibiu.ro (C.R.); liviamirelapopa@yahoo.com (M.L.P.); minodora.teodoru@ulbsibiu.ro (M.T.); oana.stoia@ulbsibiu.ro (O.S.); bogdan.neamtu@ulbsibiu.ro (B.N.); 2 County Clinical Emergency Hospital of Sibiu, 550245 Sibiu, Romania; 3 Department of Pediatric Surgery, Pediatric Hospital Sibiu, 550166 Sibiu, Romania; cristian.berghea@ulbsibiu.ro; 4 Department of Clinical Research, Pediatric Clinical Hospital Sibiu, 550166 Sibiu, Romania

**Keywords:** metabolic-associated steatotic liver disease, metabolic syndrome, atherosclerosis, noninvasive tests, vibration-controlled transient elastography, arterial stiffness, liver stiffness, pulse wave velocity, cluster analysis

## Abstract

Metabolic-associated steatotic liver disease (MASLD), previously termed non-alcoholic fatty liver disease (NAFLD), has emerged as a prominent global cause of chronic liver disease and is increasingly recognized as associated with atherosclerotic vascular illness, consolidating its position along traditional cardiovascular risk factors. Individuals with MASLD exhibit a combination of metabolic syndrome risk factors, carotid atherosclerosis, and increased arterial stiffness, hinting at shared pathogenesis. In this study, we aim to explore liver involvement and arterial stiffness within metabolic syndrome. We enrolled 75 patients (30 male and 45 female) with either liver steatosis on conventional ultrasound, altered liver function tests, or the presence of cardiometabolic risk factors after excluding liver pathology other than MASLD. Clinical evaluation, laboratory measurements, abdominal and carotid ultrasounds, vibration-controlled transient elastography (VCTE, Fibroscan), and assessment with the Arteriograph (Tensiomed) were performed. The 26 patients diagnosed with MetS had significantly higher liver involvement as quantified via the hepatic steatosis index (HSI), Fibrosis-4 (FIB4), aspartate aminotransferase to platelet ratio index (APRI) category, and VCTE measurements, as well as Agile 3+ and Agile 4 scores which use a combination of clinical and laboratory parameters together with results obtained from VCTE to reflect the probability of advanced liver fibrosis or cirrhosis. Patients with MetS also exhibited more pronounced vascular involvement as quantified via arterial stiffness measurements and CIMT (carotid intima–media thickness). We applied a two-step clustering algorithm to enhance our analysis, which gave us pertinent insight into the interplay between metabolic syndrome elements and typologies of hepatic steatosis and arterial stiffness degrees. Notably, of the three obtained clusters, the cluster showing increased levels of hepatic steatosis and arterial stiffness also exhibited the highest prevalence of metabolic syndrome and its constituting components. The results have significant clinical implications, advocating for a comprehensive diagnostic approach when MetS or MASLD is suspected.

## 1. Introduction

Metabolic-associated steatotic liver disease (MASLD), previously termed non-alcoholic fatty liver disease (NAFLD), has emerged as a prominent global cause of chronic liver disease, characterized by liver steatosis, inflammation, and fibrosis that increase morbidity and mortality [1]. Previous studies have shown the increasing burden of NAFLD, projecting a significant rise in NAFLD prevalence by 2030. As a result of this trend, the incidences of hepatic decompensation, hepatocellular carcinoma (HCC), and NASH-related cirrhosis-related deaths are also expected to increase two- to threefold by 2030 [2]. The lack of a specific pharmacological treatment and the relatively slow, asymptomatic progression of the liver disease lead to underestimation of the potential risks of morbidity and mortality in this growing population. The term NAFLD is suboptimal for characterizing the disease spectrum that spans from simple steatosis to advanced fibrosis and cirrhosis. This nomenclature falls short in encapsulating the condition’s natural progression and conveys a diagnosis of exclusion by implying “non-alcoholic,” which leaves out a substantial cohort of patients with liver steatosis when their alcohol consumption exceeds the threshold defining NAFLD. Moreover, the term “fatty” is stigmatizing, which warrants a review of terminology. Metabolic dysfunction-associated steatotic liver disease (MASLD), recently introduced to complement our understanding of hepatic steatotic diseases, has gained prominence within the medical community, but it is not used widely yet. The adoption of this updated terminology in June 2023 by major liver study societies, including the American, European, and Latin American associations, signifies a significant shift in our perception of these conditions [1]. It is important to clarify that while the terminology has evolved, certain established terms like NASH remain pertinent within this new framework. It is noteworthy that MASLD and NAFLD have prevalences that are closely aligned. From a conceptual standpoint, 98% of previously diagnosed NAFLD patients now meet the criteria for MASLD, according to an extensive analysis conducted on the European population [3]. This underscores the widespread impact of these conditions on a global scale. The newly adopted terminology emphasizes the systemic and dysmetabolic character of MASLD, which transcends beyond liver involvement. MASLD and cardiovascular disease (CVD) share risk factors, with several studies establishing MASLD as an independent CVD risk factor, contributing to 40% of MASLD-related deaths [4]. This new entity now comprises, by definition, the presence of a cardiometabolic component in addition to the traditional NAFLD criteria. The cardiometabolic criteria are identical to those used in defining metabolic syndrome, with the addition of an increased BMI.

Metabolic syndrome is defined as the clustering of three of its five defining elements according to the guidelines imposed by the European Association for the Study of the Liver (EASL), European Association for the Study of Diabetes (EASD), and European Association for the Study of Obesity (EASO) in the 2016 Clinical Practice Guidelines for the Management of Non-Alcoholic Fatty Liver Disease [5]. Those five constituents are central obesity, quantified by increased waist circumference, impaired fasting glucose or type 2 diabetes mellitus (T2DM), hypertriglyceridemia, low gender-adjusted high-density lipoprotein cholesterol, and high blood pressure. Each of these elements has a documented interaction with hepatic and vascular functionality. Regarding the connection between metabolic syndrome and MASLD, the common pathogenic mechanisms covering each of the five constituent elements of metabolic syndrome are described in detail in Appendix A—Overview of mechanisms linking MetS components to hepatic steatosis and arterial stiffness. In summary, the increase in circulating free fatty acids (FFAs) and lipid substances into liver cells initially leads to the development of hepatic steatosis, followed by cellular damage. This triggers an inflammatory response in the liver, which activates Kupffer and stellate cells, contributing to the formation of liver fibrosis. One widely accepted theory explaining the development of MASLD primarily involves insulin resistance (IR). Obesity, closely linked to IR, significantly increases the risk of MASLD, with approximately 30–90% of obese individuals developing hepatic steatosis. The progression to steatohepatitis, characterized by necroinflammation (observed in 10–20% of those with steatosis), requires additional oxidative stress.

**Figure 1:**
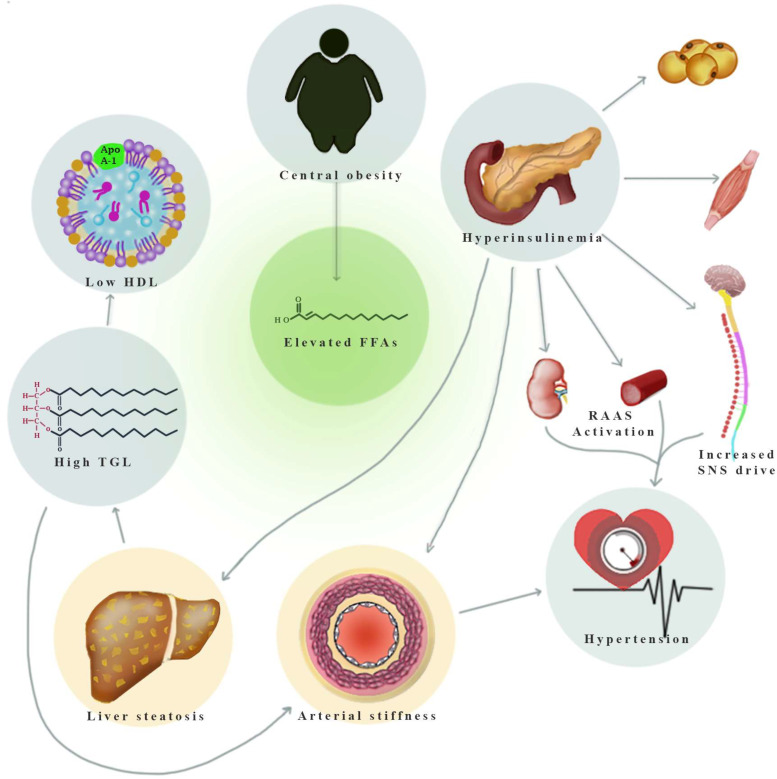
Interaction between metabolic syndrome components, liver steatosis, and arterial stiffness (explained in Appendix A—Overview of mechanisms linking MetS components to hepatic steatosis and arterial stiffness). FFAs—free fatty acids; RAAS—renin–angiotensin–aldosterone system; SNS—sympathetic nervous system; TGL—triglycerides.

Figure 1 provides a graphical representation delineating the interplay between the constituent elements of metabolic syndrome and their role in eliciting hepatic involvement and increased arterial stiffness, centered on the increased levels of free fatty acids that define this state.

Emerging evidence implies that MASLD might be linked to atherosclerotic vascular illness, further consolidating its position adjoining traditional cardiovascular risk factors. Individuals with MASLD exhibit a combination of metabolic syndrome risk factors and carotid atherosclerosis. NAFLD, in its previous definition, has been considered to be the hepatic manifestation of metabolic syndrome. The newly adopted terminology further highlights this aspect by enforcing the compulsory presence of a cardiometabolic criterion within the definition of MASLD. This diagnosis should immediately signal the presence of increased cardiovascular risk. Even though individuals with MASLD frequently fulfill the criteria for metabolic syndrome, evidence suggests a heightened risk of developing cardiovascular disease that is not solely attributed to conventional risk factors or metabolic syndrome components [6].

Recent studies additionally reveal a significant link between MASLD and increased arterial stiffness, hinting at shared pathogenesis. A review of the existing literature concerning the connections between NAFLD and aortic stiffness, with the goal of gaining deeper insights into how these two conditions interact and uncovering potential shared physiological pathways, concluded that the exact biological links between NAFLD and increased arterial stiffness are unclear, and that more prospective studies are required to explore potential causal relationships. Aortic stiffness, measured via PWV, could be a valuable tool for identifying high-risk patients for cardiovascular and liver diseases and guiding therapeutic strategies [7]. In this study, we aim to explore liver involvement and arterial stiffness within metabolic syndrome.

Arterial stiffness is a characteristic of natural vascular aging that becomes more pronounced in various cardiovascular and metabolic diseases. Significantly, arterial stiffness stands as a robust independent risk factor for an array of cardiovascular conditions, encompassing arterial hypertension, heart failure, stroke, and myocardial infarction. It is worth noting that the progression of arterial stiffening appears to occur prior to the manifestation of obvious end-organ damage. This implies that increased arterial stiffness could play a central role in connecting cardiovascular risk to subsequent diseases, making it a potential universal target for preventative and therapeutic strategies. The European Society of Cardiology’s guidelines for managing arterial hypertension recommend using pulse wave velocity (PWV) measurement to gauge arterial stiffness [8].

PWV can be measured noninvasively with tonometric, oscillometric, piezoelectric, ultrasound, and magnetic resonance techniques. The gold standard uses pressure catheters at the carotid and femoral arteries, calculating PWV from distance and transit time. Most methods estimate distance on the body surface, but this is influenced by body shape, introducing potential error. One of the methods employs time-consuming applanation tonometry for waveform collection, while the piezoelectric method demands precise catheter placement, which is particularly challenging in obese patients. All procedures are heavily dependent on operator skill. The demand for innovative and more effective diagnoses is continuously growing. There is a need to create cost-effective techniques that can serve as viable clinical solutions for early cardiovascular disease detection. The use of local versus regional assessment of PWV has been advocated in previous studies [9].

Although both PWV and the augmentation index (Aix) serve as predictors of cardiovascular risk, PWV solely represents the examined arterial segment, while the Aix is influenced by the attributes of the complete arterial system contributing to pulse wave reflection and estimates endothelial dysfunction [10].

The measurement of common carotid intima–media thickness (CIMT) using ultrasound has been suggested as a possible means of enhancing the categorization of cardiovascular risk. This is due to its direct assessment of atherosclerosis, its correlation with subsequent cardiovascular events, and its attributes of being a cost-effective, safe, and widely accessible method [11]. The assessment of carotid intima–media thickness (CIMT) offers a noninvasive, precise, and repeatable approach to detecting and measuring atherosclerotic load and cardiovascular disease risk. This method is thoroughly validated as a research instrument and is progressively finding application in clinical settings [12].

With regard to measuring liver damage, hepatic biopsy is the current gold standard in diagnosing steatotic liver disease. Its invasive nature and potential risks, however, limit its widespread application. Noninvasive tests offer a viable alternative for approximating liver involvement in MASLD. In this respect, the European Association for the Study of the Liver (EASL) advocates for the use of simple scores such as the Fib-4, the aspartate aminotransferase to platelet ratio (APRI), and the hepatic steatosis index (HSI) [13]. In addition, liver steatosis and fibrosis can reliably be assessed through vibration-controlled transient elastography (VCTE) using a Fibroscan device [14]. Current noninvasive tests for advanced fibrosis and cirrhosis detection in NAFLD patients, including APRI, FIB-4, and liver stiffness measurement via transient elastography, often yield incorrect negative predictions and false positives. Moreover, a substantial number of cases result in inconclusive findings. Combined scores using biochemical and clinical characteristics in combination with VCTE results have emerged as viable options. The Agile 3+ and Agile 4 are such scores, developed to assist in the identification of cirrhosis or advanced fibrosis among individuals with NAFLD [15]. There is a need for comprehensive assessment protocols in evaluating patients with MASLD which take into account the multisystemic involvement of this condition. To address this, one noteworthy innovation in our study lies in the utilization of machine learning techniques. By incorporating a two-step clustering algorithm, we introduce a data-driven approach that allows for a more nuanced and comprehensive analysis. This application of machine learning not only enhances our understanding of the complex interplay between metabolic syndrome elements, hepatic steatosis, and arterial stiffness but also offers a novel perspective on patient stratification. It enables us to identify distinct clusters within the study population, shedding light on previously unrecognized patterns and associations. This innovative approach has the potential to reshape how we approach and address conditions like metabolic syndrome and metabolic-associated fatty liver disease, opening new avenues for personalized diagnostics and treatment strategies.

## 2. Materials and Methods

### 2.1. Study Setting and Population

We conducted a prospective observational study in a cohort of 75 patients (30 male and 45 female), who presented for further investigation to the Sibiu Clinical County Hospital between May 2021 and May 2023 after either detection of liver steatosis on conventional ultrasound, altered liver function tests, or the presence of cardiometabolic risk factors. All the patients were clinically assessed and underwent laboratory measurements, abdominal and carotid ultrasounds, vibration-controlled transient elastography (Fibroscan), and assessment with the Arteriograph. The study was approved by the Ethics Committee, and participants provided informed consent. Patients with liver pathology other than MASLD were excluded. We screened for viral and autoimmune hepatitis, hemochromatosis, Wilson’s disease, alpha-1 antitrypsin deficiency, drug-induced liver injury, and alcohol-related liver disease. Demographic and anthropometric data were recorded.

### 2.2. Anthropometric Data

We categorized weight according to the body mass index (BMI) cut-offs given by the Center for Disease Control and Prevention [16]. Consequently, a BMI in the range 18.5–24.99 kg/m^2^ defined normal weight, 25–29.99 kg/m^2^ defined overweight, 30–34.99 kg/m^2^ was used for obesity class 1, 35–39.99 kg/m^2^ for obesity class 2, and ≥40 kg/m for obesity class 3.

### 2.3. Metabolic Syndrome Criteria, MeTS, and MASLD Diagnosis

Metabolic syndrome diagnostic criteria were defined in accordance with the European Association for the Study of the Liver (EASL), European Association for the Study of Diabetes (EASD), and European Association for the Study of Obesity (EASO) in the 2016 Clinical Practice Guidelines for the Management of Non-Alcoholic Fatty Liver Disease [5] as follows: waist circumference ≥ 94/≥80 cm for men/women (HWC), arterial pressure ≥ 130/85 mm Hg or receiving hypertension treatment (HBP), fasting glucose ≥ 100 mg/dL or under treatment for type 2 diabetes mellitus (IFG/T2DM), serum triglycerides > 150 mg/dL (HTGL), and HDL-cholesterol < 40/50 mg/dL for men/women (LHDL). The count of metabolic components in each patient (0–5) was documented, and patients with at least three criteria were diagnosed with metabolic syndrome.

MASLD requires the coexistence of at least one cardiometabolic risk factor alongside hepatic steatosis for diagnosis. The cardiometabolic risk factors coincide with the diagnostic criteria for MetS with the additional inclusion of an elevated body mass index (≥25 kg/m^2^) as an alternative cardiometabolic criterion [1].

### 2.4. Laboratory Measurements and Simple Score Calculation

Serological measurements included liver function tests, lipid profiles, fasting plasma glucose, and full blood counts. These measurements were then used to calculate the following scores:

AST/ASL ratio (AAR). AAR has been previously used with a cut-off under 0.8 for the exclusion of significant hepatic fibrosis [17].

AST to platelet ratio index (APRI). APRI has also been utilized for the exclusion of significant liver fibrosis for values under 0.5 [18]. It is calculated using the following formula (where ULN AST is the upper limit of the normal range for AST):

$$APRI=100\times \frac{\frac{AST(U/L)}{ULN\;AST(U/L)}}{PLT(10^{9}/L)}$$

FIB-4 score. A FIB-4 score under 1.3 has been used to rule out significant fibrosis [17]. It is computed using the following formula:

$$FIB4=\frac{Age\times AST(U/L)}{PLT(10^{9}/L)\times \sqrt{ALT(U/L)}}$$

Hepatic steatosis index (HSI). An HSI below 30 rules out NAFLD with a sensitivity of 93.1%, while values above 36 show a specificity of 92.4% for a positive diagnosis [19]. The following formula is used to calculate the HSI:

$$HSI=8\times \frac{ALT(U/L)}{AST(U/L)}+BMI+2\left(if\;type\;2\;diabetes\right)+2(if\;female)$$

Agile 3+ and Agile 4. The Agile scores make use of clinical and laboratory data combined with results obtained from VCTE to reflect the probability of advanced liver fibrosis or cirrhosis [15].

Agile 3+ is computed using the following formula:

$$Agile\;3+=\frac{e^{-3.92368+2.29714\times \ln E\left(kPa\right)-0.00902\times PLT\left(10^{9}/L\right)-0.98633\times \frac{ALT\left(U/L\right)}{AST\left(U/L\right)}+1.08636\times \left[Diabetes\right]-0.38581\times \left[Gender\right]+0.03018\times Age\;(y)}}{1+e^{-3.92368+2.29714\times \ln E\left(kPa\right)-0.00902\times PLT\left(10^{9}/L\right)-0.98633\times \frac{ALT\left(U/L\right)}{AST\left(U/L\right)}+1.08636\times \left[Diabetes\right]-0.38581\times \left[Gender\right]+0.03018\times Age\;(y)}}$$

And Agile 4 is computed using the following formula:

$$Agile\;4=\frac{e^{7.50139+15.42498\times \frac{1}{\surd E\left(\mathrm{kPa}\right)}-0.01378\times PLT\left(10^{9}/L\right)-1.41149\times \frac{ALT\left(U/L\right)}{AST\left(U/L\right)}-0.53281\times \left[Gender\right]+0.41741\times \left[Diabetes\right]}}{1+e^{7.50139+15.42498\times \frac{1}{\surd E\left(\mathrm{kPa}\right)}-0.01378\times PLT\left(10^{9}/L\right)-1.41149\times \frac{ALT\left(U/L\right)}{AST\left(U/L\right)}-0.53281\times \left[Gender\right]+0.41741\times \left[Diabetes\right]}}$$

### 2.5. Vibration-Controlled Transient Elastography

Fibroscan was employed to assess liver stiffness (LSM) and the controlled attenuation parameter (CAP) to quantify steatosis. Quality standards were adhered to, including overnight fasting before the examination, at least 10 measurements, and an interquartile range (IQR)/median value of LSM ≤ 0.3.

### 2.6. Arterial Stiffness Measurement

The Arteriograph employs an oscillometric method to estimate arterial stiffness. It does not directly measure the time between carotid and femoral waveforms or the distance between these recording sites. Instead, it records oscillometric pressure curves through plethysmography, capturing pressure changes in an upper arm artery. Pulsatile pressure fluctuations caused by the artery under the inflated cuff result in periodic pressure changes in the cuff. By analyzing these changes, the Arteriograph calculates the time difference between the first and reflected waves, representing the PWV distance. The device’s software decomposes systolic and diastolic waves, identifying wave onsets and peaks. It employs a cuff akin to a sphygmomanometer and calibrates using systolic and diastolic blood pressure measurements [20].

The Artheriograph (TensioMed, Budapest, Hungary) was used to measure central (aortic) systolic blood pressure (aoSBP), central (aortic) pulse pressure (aoPP), aortic augmentation index (aoAix), and aortic pulse wave velocity (aoPWV). Appropriate cuff sizes were chosen based on individual arm circumference. A single operator conducted the examinations, adhering to the manufacturer’s guidelines. A minimum of 10 min of rest was ensured before the examination. During the assessment, participants were instructed to remain still and refrain from speaking. To maintain consistency, alcohol, caffeine, and smoking were prohibited for 10 h before the examination. PWV has a normal range of 5–15 m/s, which can fluctuate based on age and blood pressure levels. Proposed reference values suggest a range of 6–10.6 m/s across various age groups. Elevated PWV is associated with reduced arterial compliance and heightened arterial stiffness [21].

### 2.7. Carotid Intima–Media Thickness

High-resolution B-mode ultrasonography was conducted using a linear array transducer of over 7 MHz frequency with minimal compression. The image plane included carotid bifurcation to ensure accurate serial measurements. CIMT was measured along a plaque-free arterial segment with a clearly defined lumen–intima and media–adventitia interface. Arterial wall segments were assessed longitudinally and perpendicular to the ultrasound beam. A straight arterial segment of 10 mm in length was selected on the far wall of the common carotid artery (CCA). The lateral probe position was chosen, which provides the best resolution for IMT measurement. CIMT measurements were taken at a location at least 5 mm below the distal end of the CCA. The reference limits for age classes 18–29, 30–39, 40–49, and 50–59 years are, respectively, 0.47, 0.59, 0.67, and 0.70 mm in women and 0.47, 0.62, 0.72, and 0.80 mm in men [22].

### 2.8. Measurement Methodology

An overview of the implemented methods to quantify the parameters regarding MetS, MASLD, arterial stiffness, and atherosclerosis is presented in Table 1.

### 2.9. Statistical Analysis

Data analysis was carried out using the IBM SPSS Statistics 21 software package. Descriptive statistics for continuous variables included calculations of mean, median, standard deviation, and 95% confidence interval, as well as minimum, maximum, and interquartile range values. Categorical variables were analyzed through frequency distributions. The Shapiro–Wilk test was employed to evaluate the normal distribution of quantitative variables. For comparing continuous variables following a normal distribution, *t*-tests were applied, while non-normally distributed variables were examined using the Mann–Whitney U test. A one-way ANOVA test was employed for mean comparison across multiple groups for continuous variables following a normal distribution within the specified groups, while the Kruskal–Wallis test was used when the data were skewed.

The chi-square or Fisher’s exact tests were employed to determine significant associations among categorical variables. Statistical significance was ascertained at a *p*-value less than 0.05.

### 2.10. Two-Step Cluster Analysis

The two-step cluster analysis technique merges K-means and hierarchical clustering methodologies to categorize observations based on shared characteristics. Multiple models were investigated, incorporating parameters obtained from VCTE and arterial stiffness measurements. A meticulous process of iterative inclusion and exclusion of variables was undertaken to refine model accuracy. Variables manifesting predictor importance below the threshold of 0.5 were deliberately omitted to enhance the model’s delineative capability. The optimal number of clusters was ascertained through a two-stage process utilizing the Akaike information criterion (AIC). An average silhouette of cohesion separation above 0.5 was considered an indicator of the good quality of the obtained model.

## 3. Results

### 3.1. Study Population and Gender Differences

A total of 75 patients (30 male and 44 female) were included in the study. There were no differences between genders regarding age, BMI, number of MetS elements, LDL-cholesterol, triglycerides, AST, FIB4, AAR, APRI, E(kPA), CAP, Agile 3+, Agile 4, AoPWV, AoAix, or CIMT. Age, HDL cholesterol, HSI, AoPWV, AoAix, and CIMT were normally distributed across genders. With regard to categorical variables, there were no differences between genders regarding the presence of MetS, T2DM, IFG, HWC, HTGL, LHDL, HBP, BMI category, APRI category, FIB4 category, AAR category, or HSI category.

Significant differences between genders are presented in Table 2.

### 3.2. Comparison between Patients with and without MetS

A total of 26 patients met the criteria for metabolic syndrome (MetS), and 49 patients did not. Age, HDL cholesterol, PLT, CAP, AoAix, CIMT, and HIS were normally distributed across MetS categories. A total of 47 patients (19 male and 28 female) were diagnosed with MASLD, while 16 had no steatosis or fibrosis as measured with the Fibroscan. The main differences between patients with MetS and those without are presented in Table 3 and Table 4. No significant differences were found between the two groups regarding platelet count, ALT, AST, LDL-cholesterol, APRI, or AAR values.

### 3.3. Cluster Analysis

Two-step cluster analysis was conducted by pairing variables from VCTE and arterial stiffness measurements. Successive model testing via exhaustive inclusion and exclusion of variables was employed. A good model was obtained when feeding the algorithm with the variables AoPWV and CAP, characterizing three clusters with an average silhouette of cohesion separation of 0.6. The general characteristics of the model and the resulting clusters are presented in Table 5. *p*-values are shown for one-way ANOVA in the case of CAP, which was normally distributed across clusters, and the Kruskal–Wallis test for AoPWV.

Cluster 1 showed significantly lower values for both parameters compared with Cluster 3, and significantly lower values for AoPWV compared with Cluster 2.

The frequency of metabolic syndrome, as well as the individual frequencies of increased weight circumference, impaired fasting glucose or type 2 DM, as well as increased blood pressure as defining characteristics of MetS, were significantly more frequent in Cluster 3, followed by Cluster 2, and were less frequent in Cluster 1. Low HDL was also more frequent in Clusters 2 and 3 compared with Cluster 1. Details are presented in Table 6.

In addition, although the categorical variable defining high triglycerides did not show significant differences in frequencies across the clusters, the absolute values of circulating triglycerides were significantly higher in Cluster 3, followed by Cluster 2, and were lower in Cluster 1, as presented in Table 7.

## 4. Discussion

Our study aims to describe liver involvement and arterial stiffness within metabolic syndrome and its constituent criteria. Of the 75 patients included, 26 fulfilled the criteria for metabolic syndrome. Results regarding the comparison between patients with and without metabolic syndrome are consistent with our previous findings [23]. Notably, in patients diagnosed with metabolic syndrome, values for age, body mass index (BMI), hepatic steatosis index (HSI), Fibrosis-4 (FIB4), Agile 3+, and Agile 4 scores, as well as E(kPa) and CAP were observed to be statistically elevated. This suggests a heightened prevalence of hepatic impairment within this cohort. In contrast to our previous findings, AST, ALT, platelet count, and the APRI score did not significantly correlate with MetS status. Nonetheless, the APRI category did correlate with the presence of MetS. There is, however, significant variance in these parameters across the presentations of MASLD/MASH and the metabolic syndrome spectrum, as previously discussed [23]. This study adds insight regarding the influence of MetS on vascular involvement, namely arterial stiffness measurements and atherosclerosis degree approximated by CIMT, and their clustering with liver damage across the same spectrum.

AoPWV, AoAix, and CIMT all showed significantly higher values in patients with MetS. These correlations have been well documented in the literature [24,25], along with their association with liver damage within MetS, as outlined above.

In our implemented clustering algorithm, three distinct clusters were identified. Notably, Cluster 3 manifested the highest CAP values as assessed with VCTE and elevated aortic pulse wave velocity (AoPWV) when juxtaposed with Cluster 1. Utilizing Cluster 1 as the referential baseline, patients classified under Cluster 3 exhibited a heightened susceptibility to both hepatic steatosis and increased arterial stiffness. Additionally, this cluster was characterized by the most prevalent occurrence of metabolic syndrome and its diagnostic criteria. Meanwhile, Cluster 2 displayed elevated arterial stiffness relative to Cluster 1 but did not exhibit significant divergences in hepatic steatosis. Consequently, Cluster 2 presented intermediate metrics between Clusters 1 and 3 concerning the prevalence of metabolic syndrome and its constituent components or their severity (namely triglycerides in the latter case). This analysis implies that individuals situated within a cluster displaying concurrent hepatic steatosis and arterial stiffness are more likely to manifest metabolic syndrome along with its diagnostic constituents. This strengthens the hypothesis that the elements of cardiometabolic dysfunction, as defined within MetS, exhibit a tendency to coalesce with both liver involvement and arterial stiffness as the severity of metabolic dysfunction escalates.

The existing literature suggests that metabolic syndrome, liver steatosis, and subclinical atherosclerosis are closely interconnected and contribute to the development of CVD [26].

Individuals with NAFLD exhibit a combination of metabolic syndrome risk factors and advanced carotid atherosclerosis. As the new terminology emphasizes, MASLD seems to be a component of metabolic syndrome, and its identification should signal the presence of increased cardiovascular risk. Even though individuals with MASLD frequently fulfill the criteria for metabolic syndrome, evidence suggests a heightened risk of developing cardiovascular disease that is not solely attributed to conventional risk factors or metabolic syndrome components.

Ozturk et al. conducted a study to assess the connection between NAFLD and subclinical atherosclerosis based on the presence or absence of metabolic syndrome. The research involved 61 individuals with NAFLD confirmed through liver biopsy and revealed that the presence of NAFLD was linked to both endothelial dysfunction and atherosclerosis, irrespective of the presence of metabolic syndrome [27].

Arterial stiffness, assessed through aortic pulse wave velocity (PWV) between the carotid and femoral arteries, serves as an indicator of forthcoming cardiovascular events. A meta-analysis of 17 studies involving over 15,000 patients established this connection by associating aortic PWV with clinical outcomes. The comparison between high and low aortic PWV groups revealed notably elevated relative risks for total cardiovascular events, cardiovascular mortality, and all-cause mortality [28].

Mechanisms such as endothelial dysfunction, altered lipid metabolism, inflammation, and insulin resistance connect MASLD to CVD, increasing the risk of issues such as hypertension, atherosclerosis, and cardiac dysfunction [29]. Despite the evidence for the connection between the two, the MASLD-CVD link is often overlooked [30].

A meta-analysis of observational studies revealed a significant association between NAFLD and an elevated risk of fatal and nonfatal cardiovascular events. However, due to the observational nature of the included studies, a definitive causal relationship between NAFLD and cardiovascular disease could not be established. Clinicians managing NAFLD patients should recognize the increased cardiovascular risk beyond liver concerns and prioritize early and proactive modification of risk factors [31].

The connection between NAFLD and CVD is driven by various factors, including endothelial dysfunction, altered lipid metabolism, inflammation, and insulin resistance, heightening the risks of hypertension, atherosclerosis, and cardiac dysfunction. This relationship has been extensively studied and reported [29].

Research suggests that liver steatosis is an independent risk factor for subclinical atherosclerosis. A 5-year longitudinal study involving 728 men and 497 women without hypertension and diabetes aimed to assess the connection between baseline non-alcoholic fatty liver disease (NAFLD) detected via ultrasound and the progression of arterial stiffness (brachial–ankle PWV). The findings revealed that individuals with NAFLD exhibited a swifter progression of arterial stiffness over time, irrespective of other cardiovascular risk factors [32].

Initiated by dyslipidemia and oxidative stress, endothelial dysfunction signifies the early phase of atherosclerosis. Research findings consistently highlight the link between coronary artery endothelial dysfunction, elevated vascular oxidative stress, prolonged atherosclerosis advancement, and augmented cardiovascular event susceptibility [33].

Inflamed visceral fat serves as a potential bridge between liver disease and atherogenesis. The liver becomes not only a target of systemic influences but also a source of proatherogenic elements. Non-alcoholic steatohepatitis contributes to cardiovascular disease by releasing inflammatory, prothrombotic, and oxidative stress molecules while also exacerbating insulin resistance and atherogenic dyslipidemia [34].

In our study, it was noted that individuals with elevated levels of liver steatosis and increased arterial stiffness had the highest occurrence of metabolic syndrome and its individual components. These results have been documented in larger cohort studies as well [35,36].

This project’s significance in terms of clinical implications is underscored by several key factors. Firstly, the project introduces a paradigm shift in how we approach liver disease within the context of metabolic syndrome. It emphasizes the need for a comprehensive evaluation when metabolic dysfunction, characteristic of metabolic syndrome, is identified. This approach recognizes that metabolic syndrome is not just a clustering of risk factors but a systemic condition with various interconnected pathologies. By extending this comprehensive evaluation to include liver assessment, we acknowledge the pivotal role of the liver in this systemic dysmetabolic condition. This recognition is crucial for identifying and managing potential liver-related complications effectively.

Secondly, the adoption of new terminology, specifically the term “MASLD”, represents a substantial improvement over the previous label of “non-alcoholic” liver disease. By focusing on the metabolic aspects of the condition, this terminology provides a more accurate and holistic description. Importantly, it avoids the negative connotations and stigma associated with the diagnosis of exclusion. This shift in terminology not only reflects the underlying pathophysiology more accurately but also fosters a more positive and empathetic approach to patient care.

Furthermore, establishing a standardized definition for MASLD has far-reaching benefits. It streamlines diagnosis, aids in conducting research, and facilitates the development of effective management strategies. Importantly, it allows for the continuity and validation of previous research efforts, particularly in the context of steatohepatitis (NASH). The clinical definition of steatohepatitis remains relevant within the framework of MASLD, ensuring that the wealth of existing data and insights from previous studies can continue to inform the care of individuals with this condition.

The term MASLD also enhances patient understanding. It links liver disease directly to underlying cardiometabolic disorders, particularly insulin resistance and its associations with other conditions. This linkage helps patients grasp the broader context of their condition and its systemic implications, moving away from the perception of a diagnosis of exclusion.

Moreover, the project’s approach facilitates efficient communication among healthcare providers. It enables clear discussions about the necessary therapeutic measures, from both a liver-specific perspective and a holistic view that addresses cardiometabolic health. Additionally, aligning the diagnostic criteria for MASLD with well-established phenotypic characteristics in diabetology and cardiology simplifies the identification of patients with this condition. This alignment enhances clinical practice by making it easier for physicians to identify and manage patients with MASLD, leading to more effective care and potentially improving outcomes.

In summary, this project’s significance in clinical implications lies in its holistic approach to liver disease within the context of metabolic syndrome, the introduction of improved terminology, the establishment of standardized definitions, enhanced patient understanding, and improved communication and disease awareness among healthcare providers. These factors collectively contribute to more effective diagnosis and management of individuals with MAFLD, addressing the complexities of liver disease within the broader context of metabolic syndrome.

### 4.1. Strengths and Limitations

The limitations of the current investigation predominantly pertain to the modest sample size employed, as well as the absence of gold-standard techniques for liver assessment (hepatic biopsy) and PWV measurement (catheterization), both of which are invasive in nature, however.

Notwithstanding, it is worth noting that the oscillometric noninvasive method has demonstrated ease of use and validity in estimating hemodynamic variables like central and peripheral arterial pressure. In a study of 100 patients undergoing left cardiac catheterization it exhibited strong agreement and conformity with the gold standard across diverse patient types and conditions. This technique can potentially enhance cardiovascular assessment in primary and secondary prevention, optimize treatment for specific patients, and offer valuable insights for future cardiovascular prevention strategies [37].

Despite the constraints outlined above, our methodological approach and resultant findings are in alignment with extant literature that has employed comparable sample sizes [38]. Furthermore, although our study did not utilize hepatic biopsy to objectively evaluate liver involvement among the participating patients, it is worth noting that the deployment of noninvasive assessments is endorsed by the European Association for the Study of the Liver for the evaluation of MASLD [13]. The ease of implementation and widespread availability of such noninvasive techniques imbue our study with clinically relevant practical implications.

Of particular note is the novel approach utilizing a two-step clustering algorithm to delineate between different populations within the MetS spectrum with regard to liver steatosis and arterial stiffness, offering accurate and easy-to-interpret results.

### 4.2. Future Directions

Future research should focus on refining our understanding of the intricate association between metabolic syndrome and its multiorgan ramifications. Specifically, the identification of abnormalities in a singular aspect of the metabolic syndrome spectrum should prompt rigorous scrutiny for a potential underlying systemic pathology that is emblematic of metabolic syndrome as a whole. This comprehensive approach should be expanded to include hepatic dysfunction, as it serves as a critical indicator of the systemic characteristics inherent in this dysmetabolic condition.

The search for dietary and lifestyle approaches that have demonstrated efficacy in hindering the vicious cycles associated with metabolic syndrome [39] merits prioritized attention in preventing MetS-associated cardiovascular risk. Diligence in promoting body fitness should accompany this effort, as underscored by an extensive body of evidence, including animal model studies, that have established a connection between insulin resistance and a lack of sustained physical activity [40].

Concurrently, the complex impact of global phenomena, such as the COVID-19 pandemic, may still warrant investigation, possibly elucidating how social engagement plays a role in lifestyle alteration [41].

Future studies on larger sample sizes could further enhance the results obtained by implementing machine learning algorithms, which would present the opportunity of developing large-scale prediction models.

## 5. Conclusions

Our study aimed to investigate the relationship between metabolic syndrome (MetS), liver dysfunction, and arterial stiffness. Out of 75 patients, 26 met the criteria for MetS and exhibited statistically elevated markers indicating hepatic impairment and arterial stiffness. The study’s two-step cluster analysis identified three clusters that varied in the severities of MetS, hepatic steatosis, and arterial stiffness, supporting the link between these health issues. These findings bolster the hypothesis that elements of cardiometabolic dysfunction in MetS often occur in conjunction with liver involvement and arterial stiffness. The results suggest a need for multiorgan scrutiny when MetS is suspected and hint towards the role of lifestyle and dietary interventions in MetS management. Future research should further explore the systemic pathology underlying MetS.

## Figures and Tables

**Table 1:** Methods overview.

Aspect Measured	Parameter	Device	Method	Cut-Offs
Weight status	BMI	Electronic scale, stadiometer	A calibrated scale was used to measure the patient’s body weight in kilograms (kg). In order to minimize measurement errors, lightweight clothing and no shoes were worn. Height in centimeters (cm) was measured while the patient was standing erect with their back against a wall and heels together. The following formula was employed: BMI = Weight (kg)/(Height m^2^)	Underweight: BMI less than 18.5 Normal weight: BMI 18.5 to 24.9 Overweight: BMI 25 to 29.9 Obesity class 1: BMI 30 to 34.9 Obesity class 2: BMI 35 to 39.9 Obesity class 3: BMI 40 or greater
MetS	WC	Flexible measuring tape	The measuring tape was positioned around the waistline, horizontal and parallel to the floor. It was wrapped snugly around the waist without compressing the skin, and not over clothing. The measurement was taken in centimeters.	≥94/≥80 cm for men/women
	Blood pressure	Manual sphygmomanometer; stethoscope	At least two measurements taken, using the appropriate cuff size, after the patient rested in a seated position for at least 5 min. The results were expressed in mmHg.	≥130/85 mm Hg
	Fasting plasma glucose	Automated analyzer	A fast of at least 8 h before the test. The blood sample was collected through venipuncture and labeled with the individual’s information, including name and date of birth, to ensure accurate identification. The sample was sent to the laboratory for analysis. The result was reported in milligrams per deciliter (mg/dL).	≥100 mg/dL
	HDL Cholesterol		Blood was drawn after a minimum 8 h fast, and the sample was adequately labeled and sent to the laboratory. The result was reported in milligrams per deciliter (mg/dL).	<40/50 mg/dL for men/women
	Serum Triglycerides			>150 mg/dL
Arterial stiffness	PWV, AiX	Arteriograph Tensiomed (Medexpert, Budapest). Software version 3.0.0.3	Participants refrained from alcohol, caffeine, and smoking for at least 10 h before the examination. After 10 min of rest in a supine position, measurements were taken. During the assessment, patients were instructed to minimize movement and remain quiet to reduce interference. Appropriate cuff sizes, determined by individual arm circumference using a flexible measuring tape, were selected. Additionally, the distance from the jugular notch to the symphysis pubis was measured to estimate travel distance. Demographic data, including age, gender, height, weight, and smoking status, were recorded. An appropriately sized cuff, similar to a sphygmomanometer, was placed on the patient’s arm. The device then automatically inflated and gradually deflated the cuff while communicating with a laptop. This method records oscillometric pressure curves through plethysmography, capturing pressure changes in an upper arm artery. Pulsatile pressure fluctuations caused by the artery under the cuff result in periodic pressure changes. The Arteriograph calculates PWV and Aix by analyzing these changes, decomposing systolic and diastolic waves, and identifying wave onsets and peaks using its software.	Assessed as continuous variables
Atherosclerosis	CIMT	General Electric S8 ultrasound device, using a >7 MHz linear array transducer	High-resolution B-mode ultrasonography was conducted with minimal compression. CIMT assessments were made on arterial segments free of plaques, characterized by clear lumen–intima and media–adventitia interface. A straight 10 mm long segment of the far wall of the common carotid artery (CCA) was selected. The specific region of interest was expanded to a high-resolution 1.2 × 1.2 cm image. Longitudinal images of the carotid arteries were captured using the lateral probe position, which optimizes CIMT resolution. CIMT measurements were taken at a location situated at least 5 mm below the distal end of the CCA.	Assessed as continuous variable
Liver involvement	Platelet count, AST, and ALT for simple score calculation	Automated analyzer	Blood samples were collected after an overnight fast through venipuncture using appropriate collection tubes.	HIS, FIB4, APRI, AAR cut-offs described in text
	E (kPa), CAP	Fibroscan 502 Touch	The patient undergoes a three-hour fasting period before the procedure and is positioned in a supine posture with the right arm abducted. The choice between the M and XL probe is determined based on the device’s prompt for optimal assessment. The method of 1D transient elastography provides a quantitative assessment of 1D elasticity within hepatic tissue, referred to as liver stiffness measurement (LSM) or E (measured in kPa). This method also enables the measurement of steatosis using the controlled attenuation parameter (CAP, measured in dB/m). It involves the generation of a mechanical pulse to estimate tissue stiffness along a fixed ultrasonographic line. The assessed tissue volume is approximately 1 cm × 4 cm. To ensure accuracy, ten measurements were taken, and the variability between these measurements was less than 30% of the mean stiffness value (interquartile range, IQR).	Assessed as continuous variables

**Table 2:** Differences between genders.

Variable	Descriptive Parameter	Gender		*p*-Value
		Female	Male	
AST (U/L)	Mean	24.13	33.87	0.045
	StdDev	13.18	36.72	
	IQR	9	14	
	MIN	12	10	
	MAX	82	217	
	95% CI	20.17–28.09	20.16–47.58	
Platelets (10^9^/L)	Mean	280.07	252.9	0.044
	StdDev	83.93	63.72	
	IQR	102	65.75	
	MIN	53	154	
	MAX	480	441	
	95% CI	254.85–305.28	229.11–276.69	
APRI	Mean	0.3440	0.4060	<0.01
	StdDev	0.4978	0.3673	
	IQR	0.12	0.2	
	MIN	0.08	0.07	
	MAX	3.16	2.06	
	95% CI	0.1944–0.4936	0.2689–0.5431	
HDL-cholesterol	Mean	65.2	54.93	<0.01
	StdDev	15.66	12.73	
	IQR	19.5	20.75	
	MIN	37	34	
	MAX	100	80	
	95% CI	60.5 –69.9	50.18–59.69	
HSI	Mean	40.99	37.10	<0.01
	StdDev	6.32	5.01	
	IQR	9.97	8.53	
	MIN	26.8	27.77	
	MAX	53.37	46.18	
	95% CI	39.09–42.89	35.23–38.98	

**Table 3:** Comparison between patients with and without MetS—continuous variables.

Variable	Descriptive Parameter	MetS		*p*-Value
		No	Yes	
Age	Mean	50.92	66.81	<0.01
	StdDev	13.17	11.03	
	IQR	20	16	
	MIN	19	45	
	MAX	74	85	
	95% CI	47.14–54.7	62.35–71.26	
BMI	Mean	26.88	31.49	<0.01
	StdDev	3.99	5.05	
	IQR	5.2	7.83	
	MIN	20.8	24.2	
	MAX	40.6	42.7	
	95% CI	25.73–28.02	29.45–33.53	
HDL-cholesterol	Mean	65.3	53.15	<0.01
	StdDev	14.06	14.68	
	IQR	17.5	23	
	MIN	38	34	
	MAX	100	92	
	95% CI	61.27–69.35	47.22–59.08	
Triglycerides	Mean	105.63	152.04	<0.01
	StdDev	57.11	75.54	
	IQR	53	75.75	
	MIN	26	42	
	MAX	293	368	
	95% CI	89.23–122.04	121.53–182.55	
HSI	Mean	37.92	42.31	<0.01
	StdDev	5.99	5.32	
	IQR	9.33	7.34	
	MIN	26.8	31.3	
	MAX	51.3	53.37	
	95% CI	36.19–39.64	40.16–44.46	
FIB-4	Mean	1	1.93	<0.01
	StdDev	0.73	2.23	
	IQR	0.5	1.1	
	MIN	0.22	0.42	
	MAX	4.4	11.49	
	95% CI	0.79–1.21	1.02–2.83	
Agile 3+	Mean	0.128	0.4509	<0.01
	StdDev	0.1597	0.3194	
	IQR	0.1354	0.5699	
	MIN	0.0095	0.0382	
	MAX	0.9082	0.9915	
	95% CI	0.0821–0.1738	0.3219–0.58	
Agile 4	Mean	0.0232	0.131	<0.01
	StdDev	0.0982	0.2283	
	IQR	0.0121	0.1641	
	MIN	0.0003	0.0003	
	MAX	0.6921	0.8459	
	95% CI	−0.005–0.0514	0.0388–0.2233	
CAP	Mean	255.22	303.92	<0.01
	StdDev	51.18	49.56	
	IQR	81	52.5	
	MIN	140	153	
	MAX	361	381	
	95% CI	240.52–269.93	283.9–323.94	
E(kPa)	Mean	4.86	8.97	<0.01
	StdDev	1.96	5.96	
	IQR	1.5	5.83	
	MIN	2.4	2.1	
	MAX	15.6	27.7	
	95% CI	4.29–5.42	6.57–11.37	
AoPWV	Mean	8.87	9.73	0.029
	StdDev	2.54	1.33	
	IQR	3.65	2.03	
	MIN	4.1	7.6	
	MAX	17.7	13.3	
	95% CI	8.14–9.6	9.19–10.26	
AoAix	Mean	29.12	38.78	<0.01
	StdDev	15.44	13.89	
	IQR	22.45	20.58	
	MIN	−2.3	11.4	
	MAX	62.8	70.8	
	95% CI	24.68–33.55	33.17–44.39	
CIMT	Mean	0.74	1.05	<0.01
	StdDev	0.19	0.13	
	IQR	0.3	0.23	
	MIN	0.4	0.8	
	MAX	1.2	1.3	
	95% CI	0.69–0.8	1–1.11	

**Table 4:** Comparison between patients with and without MetS—categorical variables.

Variable	Values	MetS		*p*-Value
		No	Yes	
BMI category	Normal	23 (46.9%)	2 (7.7%)	<0.01
	Overweight	15 (30.6%)	9 (34.6%)	
	Obese (class 1)	9 (18.4%)	8 (30.8%)	
	Obese (class 2)	1 (2%)	5 (19.2%)	
	Obese (class 3)	1 (2%)	2 (7.7%)	
APRI ≥ 0.5	No	47 (95.9%)	20 (76.9%)	0.018
	Yes	2 (4.1%)	6 (23.1%)	
FIB-4 ≥ 1.3	No	43 (87.8%)	13 (50%)	<0.01
	Yes	6 (12.2%)	13 (50%)	
HSI > 36	No	19 (38.8%)	4 (15.4%)	0.037
	Yes	30 (61.52%)	22 (84.6%)	

**Table 5:** Two-step cluster model overview.

Variable	Characteristic	Cluster 1	Cluster 2	Cluster 3	*p*-Value
Count	-	28 (37.3%)	13 (17.3%)	34 (45.3%)	
CAP (dB/m)	Mean	226.46	246.23	319.58	<0.01
	StdDev	39.33	36.57	39.33	
	IQR	58.25	33.5	45.25	
	MIN	140	198	271	
	MAX	300	336	381	
	95% CI	211.21–241.71	224.13–268.33	309.81–326.37	
	Predictor importance	1			
AoPWV	Mean	7.38	12.23	9.46	<0.01
	StdDev	1.43	2.02	1.2	
	IQR	2.03	2.2	1.78	
	MIN	4.1	9.8	7.6	
	MAX	10.1	17.7	12	
	95% CI	6.83–7.94	11–13.45	9.05–9.88	
	Predictor importance	0.88			

**Table 6:** MetS and constituent elements across clusters.

Variable	Values	Cluster 1	Cluster 2	Cluster 3	*p*-Value
MetS	No	25 (89.3%)	10 (76.9%)	14 (41.2%)	<0.01
	Yes	3 (10.7%)	3 (23.1%)	20 (58.8%)	
HWC	No	26 (92.9%)	8 (61.5%)	11 (32.4%)	<0.01
	Yes	2 (7.1%)	5 (38.5%)	23 (67.6%)	
IFG/T2DM	No	19 (67.9%)	7 (53.8%)	8 (23.5%)	<0.01
	Yes	9 (32.1%)	6 (46.2%)	26 (76.5%)	
LHDL	No	27 (96.4%)	9 (69.2%)	24 (70.6%)	0.023
	Yes	1 (3.6%)	4 (30.8%)	10 (29.4%)	
HBP	No	22 (78.6%)	5 (38.5%)	11 (32.4%)	<0.01
	Yes	6 (21.4%)	8 (61.5%)	23 (67.6%)	

**Table 7:** Triglycerides across clusters.

Variable	Characteristic	Cluster 1	Cluster 2	Cluster 3	*p*-Value
Triglycerides	Mean	96.96	120.85	142.44	<0.01
	StdDev	55.42	60.28	73.33	
	IQR	46.75	45.5	59.25	
	MIN	26	65	42	
	MAX	293	292	368	
	95% CI	75.48–118.45	84.42–157.27	116.85–168.03	

## Data Availability

The data presented in this study are available upon reasonable request from the corresponding author.

## Appendix A. Overview of Mechanisms Linking MetS Components to Hepatic Steatosis and Arterial Stiffness

### Appendix A.1. Central Obesity

There is a well-established link between obesity and cardiovascular disease. Central obesity, in particular, augments the risk associated with increased adiposity. Hypertrophic adipocytes have altered lipolytic capabilities in response to insulin and show several maladaptive patterns related to normal adipokine secretion. These aspects are further discussed in a recent review conducted by part of our team, highlighting the mechanisms leading from childhood and adolescent obesity to adult cardiovascular disease [42]. Of particular interest in the context of the interplay between central obesity as a metabolic syndrome component and MASLD, as well as increased arterial stiffness and atherosclerosis, is the increase in free fatty acids (FFAs) mediated by dysfunctional hypertrophic adipocytes, which are incapable of adequate lipolysis. The resulting excess FFAs are released into the bloodstream and delivered throughout the organism [43]. This results in the activation of a range of either adaptive or maladaptive physiological mechanisms in peripheral tissues, specifically designed to mitigate the surfeit of free fatty acids (FFAs). Such a phenomenon is emblematic of a multiorgan response to elevated lipid influx [44].

Within the liver, the surplus in FFAs may exceed the capabilities of conventional metabolization in the mitochondria, leading to a shift towards extramitochondrial beta-oxidation with a subsequent increase in intracellular reactive oxygen species. The process of diverting free fatty acids eventually involves toxic metabolite formation, such as ceramide formation and excess intrahepatic lipid storage [45]. Lipogenesis and gluconeogenesis are also engaged as metabolic pathways to direct the excess in FFAs [46]. All these mechanisms are staples of liver affliction in MASLD.

Two principal theories attempt to explain how central obesity leads to increased FFAs. The “portal theory” stipulates that excessive central adipose tissue leads to direct hepatic exposure to increased local FFA production by means of the portal vein. This would then exert the effects mentioned above within the hepatocytes, leading to increased local insulin resistance with a consequent increase in hepatic glucogenesis. Eventually, this leads to a systemic hyperglycemic state with increased insulin production in response, which, in time, elicits an attenuated peripheral effect in the form of insulin resistance [47]. The “spillover theory,” on the other hand, stipulates that when chronically and excessively exposed to an elevated caloric status, the subcutaneous compartment of adipose tissue can no longer expand to accommodate the organism’s need to store lipid deposits. This would determine a “spillover” of free fatty acids from within the subcutaneous adipose compartment towards visceral fat and non-adipose tissues—including the liver [48].

With regard to the vascular impact of increased free fatty acids, a relationship has been documented between this increase and arterial stiffness [49]. Several mechanisms have been proposed to contribute to this aspect, including a decrease in nitric oxide synthase activity and endothelium-mediated vascular tonus regulation [50], as well as increased reactive oxygen species production, with the latter being a hallmark of inflammation, fibrosis, microvascular tonus, and remodeling disruption [51].

With these aspects in consideration, adipose tissue, which emerges in the current view as a standalone endocrine organ [52], when disrupted in its normal functioning, may play a quintessential role in triggering hepatic and vascular modifications associated with MASLD.

### Appendix A.2. Hypertriglyceridemia

Hypertriglyceridemia is yet another definitory aspect of the dysmetabolic processes encompassed by metabolic syndrome. As previously described, exposure to increased free fatty acids leads to the activation of various pathways attempting to manage and metabolize the excess. The formation of different lipid compounds such as ceramides, diacylglycerols, and triglycerides is one such direction. There is a known propensity towards hypertriglyceridemia in liver steatosis [53], and it has been proposed that the link between increased arterial stiffness and MASLD may, in fact, be governed by an increase in circulating triglyceride levels [7,49].

Unlike cholesterol, triglycerides do not directly accumulate within atheromatous plaque but exert an indirect atherogenic effect. This can occur either by reducing high-density lipoprotein (HDL) cholesterol levels or by fostering the buildup of lipoproteins that possess intrinsic atherogenic potential.

The mechanisms underlying this indirect atherogenic impact are threefold. Firstly, an escalation in triglyceride-enriched and cholesterol-depleted low-density lipoprotein (LDL) particles emerges, leading to alterations in particle density and size. This transformation renders them smaller, enhancing their ability to permeate the arterial wall, become ensnared by proteoglycans in the subendothelial space, and undergo oxidation. Secondly, hypertriglyceridemia triggers the accumulation of very-low-density lipoprotein (VLDL) and chylomicron remnants, which, due to their size, penetrate the subendothelial space, mirroring the behavior of LDL and contributing to the development of atheromatous plaque. Lastly, excessive production of VLDL heightens competition with chylomicrons for lipoprotein lipase activity, leading to the accumulation of the latter during fasting periods [54].

### Appendix A.3. Low HDL-Cholesterol

The circumvention of lipid metabolism towards cholesterol ester and triglyceride formation in the context of excessive free fatty acids leads to an increase in hepatic VLDL production to mitigate the additional lipid compounds. VLDL then interacts with HDL by means of the cholesterol ester transfer protein, thus speeding the degradation of HDL. These mechanisms provide the basis for the typical atherogenic lipid profile describing metabolic syndrome patients with increased triglycerides and low HDL [55].

HDL’s antiatherogenic properties are exemplified by its capacity to facilitate reverse cholesterol transport (RCT), a process critical for removing cholesterol from peripheral tissues to the liver for excretion. Despite this protective mechanism, conditions marked by insulin resistance and systemic inflammation compromise HDL’s abilities, transforming it into proinflammatory particles [56]. These changes have been attributed to alterations in the composition of HDL proteins, with over 80 less common HDL proteins identified alongside Apolipoprotein AI and Apolipoprotein AII. These HDL proteins play roles in lipid metabolism, the acute-phase response, and innate immunity, collectively contributing to HDL’s anti-inflammatory and antiatherogenic functions and determining HDL’s intricate roles in lipid homeostasis and cardiovascular health [57].

Patients diagnosed with MASLD frequently exhibit diminished levels of high-density lipoprotein cholesterol (HDLc), a well-established indicator of cardiovascular disease (CVD) risk [58].

### Appendix A.4. Impaired Fasting Glucose/T2DM

Impaired fasting glucose is the precursor state to type 2 diabetes, sharing the same pathways rooted in the development of insulin resistance. Increased FFAs may play a role in this regard by influencing pancreatic local inflammatory status and directly mediating beta-cell insulin secretion [43]. In essence, the well-documented mechanism centered around chronic hyperglycemia, which leads to increased insulin production and subsequent reduced peripheral insulin sensitivity, forming a vicious cycle, is interrelated with hepatic steatosis and arterial stiffening [7]. In this context, both hyperglycemia and insulin resistance have been linked to increasing arterial stiffness by disrupting vascular smooth muscle cell function and proliferation, as well as the extracellular matrix composition within the arterial wall [24]. In addition, MASLD-attributable liver dysfunction pertaining to altered hepatic insulin metabolism further perpetuates the vicious cycle of hyperglycemia, hyperinsulinemia, and insulin resistance. Subsequently, these elements trigger additional inappropriate management of lipid components such as excess FFAs, in turn aggravating liver damage [59].

### Appendix A.5. Hypertension

Of all the five defining criteria of metabolic syndrome, arterial hypertension seems to be the most intuitively linked to increased arterial stiffness, as this phenomenon provides the basis for increased systolic and decreased diastolic blood pressure with age [60]. The pathways involved in generating an increase in arterial stiffness and circulating blood pressure have shared roots with those leading to hepatic involvement within MetS. Long-term exposure to hyperinsulinemia and hyperglycemia has been shown to augment renin–angiotensin–aldosterone system activation and stimulate vascular angiotensin type 1 (AT1) receptor expression. This, in turn, induces vascular wall thickening due to vascular smooth muscle cell proliferation and promotes vasoconstriction and vascular fibrosis, all of which are key mechanisms within arterial stiffening and blood pressure regulation [7,61,62]. Hyperinsulinemia also leads to increased sympathetic drive, a quintessential factor involved in the pathogenesis of hypertension. Increased insulin levels exert their stimulatory action by direct central activation of the sympathetic nervous system. Yet another vicious cycle ensues in this regard, whereby increased sympathetic tonus leads to increased insulin levels [63].
